# Association between autoimmune diseases and risk of developing breast cancer-related lymphedema

**DOI:** 10.1038/s44483-026-00004-3

**Published:** 2026-10-24

**Authors:** Benjamin D. Wagner, Stav Brown, Andrea V. Barrio, Thomas Amburn, Natália Polidorio, Tiana Le, Lillian A. Boe, Raghu P. Kataru, Geoffrey E. Hespe, Michelle R. Coriddi, Danielle H. Rochlin, Hidehiko Yoshimatsu, Babak J. Mehrara

**Affiliations:** 1 Department of Surgery, Plastic and Reconstructive Surgery Service, Memorial Sloan Kettering Cancer Center, New York, NY USA; 2 Department of Surgery, Breast Service, Memorial Sloan Kettering Cancer Center, New York, NY USA; 3 Department of Epidemiology and Biostatistics, Biostatistics Service, Memorial Sloan Kettering Cancer Center, New York, NY USA

**Keywords:** Cancer, Diseases, Immunology, Medical research, Rheumatology, Risk factors

## Abstract

Preexisting autoimmune disease may increase susceptibility to breast cancer-related lymphedema (BCRL) through chronic inflammation and immune dysregulation, but its association with BCRL risk is poorly understood. We conducted a retrospective cohort study of adults undergoing sentinel lymph node biopsy and/or axillary lymph node dissection for breast cancer at a tertiary cancer center between 2010 and 2021. Autoimmune diseases were identified using International Classification of Diseases (ICD)-9/10 codes, and the primary outcome was BCRL diagnosis based on ICD codes. Associations between autoimmune disease and time to BCRL were evaluated using univariate and multivariable Cox proportional hazards models. Among 20,027 patients with a median follow-up of 61.7 months (IQR, 38.7–94.2), 1361 (6.8%) had a preexisting autoimmune disease and 1728 (8.6%) developed BCRL at a median of 17.4 months (IQR, 8.0–33.9) after surgery. In multivariable analysis, autoimmune disease was independently associated with increased BCRL risk (hazard ratio [HR], 1.52; 95% CI, 1.30–1.77; *P* < .001). Elevated risks were observed for rheumatoid arthritis (HR, 1.54), autoimmune thyroiditis (HR, 1.40), Graves’ disease (HR, 1.49), multiple sclerosis (HR, 1.87), systemic lupus erythematosus (HR, 2.46), and systemic sclerosis (HR, 2.10). These findings suggest autoimmune disease may be an independent risk factor for BCRL and may improve risk stratification.

## Introduction

Breast cancer-related lymphedema (BCRL) is the most common form of secondary lymphedema in Western countries and represents a major source of morbidity among breast cancer survivors.^1^ Pooled estimates suggest that approximately 20% of patients develop lymphedema following breast cancer treatment.^2^ However, the reported incidence of BCRL varies widely, from less than 5% to more than 50%, depending on treatment modality, method of diagnosis, and duration of follow-up.^2–4^ BCRL is characterized by chronic inflammation, progressive swelling of the affected extremity, fibroadipose tissue deposition, and, in some patients, recurrent infections.^5^ These pathologic changes frequently result in pain, functional limitations, and diminished quality of life.^6^

The development of BCRL is multifactorial, reflecting the interplay of treatment-related, clinical, and patient-specific factors. Established risk factors include axillary lymph node dissection (ALND), adjuvant radiotherapy to the breast or regional lymph nodes, taxane-based chemotherapy, and obesity.^7–11^ Racial and ethnic disparities have also been observed, with Black and Hispanic women more likely to develop BCRL and experience more severe disease.^10–12^ In addition to these clinical associations, recent preclinical and translational studies have highlighted the role of chronic inflammation in the pathogenesis of secondary lymphedema. Activation of the arachidonic acid pathway, leukotriene signaling, neutrophil and macrophage infiltration, and T-helper 2 (Th2)-driven immune responses have been implicated in the regulation of fibrofatty tissue deposition, epidermal changes, susceptibility to infections, and lymphatic vessel remodeling.^13–19^ T-cell responses are oligoclonal and require dendritic cell activation, migration, and antigen presentation in regional lymph nodes.^16,20,21^ Notably, T-cell clones with autoreactivity to insulin and other self-antigens have been identified in specimens from patients with BCRL, suggesting that autoimmune mechanisms may contribute to disease pathogenesis.^16^ This concept is further supported by observations that patients with autoimmune diseases such as systemic lupus erythematosus (SLE) and rheumatoid arthritis (RA) also exhibit lymphatic dysfunction, impaired lymph clearance, and increased cutaneous inflammation.^22,23^

To our knowledge, no previous study has analyzed the association between autoimmune diseases and the risk of BCRL development. Therefore, the aim of this study was to use a large institutional database of patients treated for breast cancer and analyze autoimmune diseases as an independent risk factor for BCRL development. We hypothesized that patients with preexisting autoimmune disease would have an increased risk of developing BCRL than those without, even after adjustment for clinical and treatment-related factors.

## Results

A total of 20,027 patients were included, with a median (IQR) follow-up of 61.7 (38.7–94.2) months. Most patients were female (19,852 [99%]) and self-identified as White (14,081 [70%]); 1724 (8.6%) were Asian, 1783 (8.9%) Black, 1393 (7.0%) Hispanic, 265 (1.3%) other, and 781 (3.9%) unknown race or ethnicity. The median (IQR) age at axillary surgery was 55 (47–65) years, and the median BMI was 25.0 (21.9–29.2) kg/m^2^. Among the cohort, 3791 (19%) patients underwent ALND and 16,236 (81%) underwent SLNB. Breast-conserving surgery was performed in 12,209 (61%) patients and mastectomy in 7816 (39%). Chemotherapy was received by 10,461 (52%) patients, including 6432 (32%) in the adjuvant setting and 3871 (19%) in the neoadjuvant setting. Radiotherapy was administered to 13,139 (66%) patients, including 2723 (14%) who received nodal irradiation. Endocrine therapy was received by 15,406 (77%) patients. Baseline demographic, clinical, and treatment characteristics are summarized in Table 1.

**Table 1:** Baseline demographic and clinical characteristics of the study cohort overall and by breast cancer-related lymphedema (BCRL) diagnosis

Characteristic	Overall (*n* = 20 027)	BCRL (*n* = 1728)	No BCRL (*n* = 18 299)
Age at surgery, median (IQR), years	55.0 (47.0–65.0)	51.0 (44.5–61.0)	56.0 (47.0, 65.0)
Sex, No. (%)			
Female	19 852 (99)	1712 (99)	18 140 (99)
Male	175 (0.9)	16 (0.9)	159 (0.9)
Race/ethnicity, No. (%)			
Asian	1724 (8.6)	120 (6.9)	1604 (8.8)
Black	1783 (8.9)	252 (15)	1531 (8.4)
Hispanic	1393 (7.0)	152 (8.8)	1241 (6.8)
Other	265 (1.3)	30 (1.7)	235 (1.3)
Unknown	781 (3.9)	68 (3.9)	713 (3.9)
White	14,081 (70)	1106 (64)	12,975 (71)
BMI, median (IQR), kg/m^2^	25.0 (21.9–29.2)	25.7 (22.2–29.9)	25.0 (21.9–29.1)
Smoking status, No. (%)			
Current smoker	481 (2.4)	47 (2.7)	434 (2.4)
Former smoker	3325 (17)	223 (13)	3102 (17)
Never smoker	13,600 (68)	1253 (73)	12,347 (67)
Unknown	2621 (13)	205 (12)	2416 (13)
Axillary surgery, No. (%)			
ALND	3791 (19)	1057 (61)	2734 (15)
SLNB	16,236 (81)	671 (39)	15,565 (85)
Breast surgery, No. (%)			
BCS	12,209 (61)	696 (40)	11,513 (63)
Mastectomy	7816 (39)	1031 (60)	6785 (37)
No breast surgery	2 (<0.1)	1 (<0.1)	1 (<0.1)
Any chemotherapy, No. (%)			
No	9562 (48)	244 (14)	9318 (51)
Unknown	4 (<0.1)	0 (0)	4 (<0.1)
Yes	10461 (52)	1484 (86)	8977 (49)
Chemotherapy timing, No. (%)			
Adjuvant	6432 (32)	687 (40)	5745 (31)
Neoadjuvant	3871 (19)	749 (43)	3122 (17)
None	9562 (48)	244 (14)	9318 (51)
Unknown	162 (0.8)	48 (2.8)	114 (0.6)
Any radiotherapy, No. (%)			
No	6249 (31)	256 (15)	5993 (33)
Unknown	639 (3.2)	18 (1.0)	621 (3.4)
Yes	13,139 (66)	1454 (84)	11,685 (64)
Nodal radiotherapy, No. (%)			
No	16,629 (83)	1109 (64)	15,520 (85)
Unknown	675 (3.4)	19 (1.1)	656 (3.6)
Yes	2723 (14)	600 (35)	2123 (12)
Any endocrine therapy, No. (%)			
No	4327 (22)	405 (23)	3922 (21)
Unknown	294 (1.5)	6 (0.3)	288 (1.6)
Yes	15,406 (77)	1317 (76)	14,089 (77)
Follow-up, median (IQR), months	61.7 (38.7–94.2)	76.6 (50.6–110.0)	60.8 (37.9–92.3)

*ALND* axillary lymph node dissection, *BCS* breast-conserving surgery, *BMI* body mass index, *IQR* interquartile range, *SLNB* sentinel lymph node biopsy.

Overall, 1728 (8.6%) patients developed BCRL, with a median (IQR) time to onset of 17.4 (8.0–33.9) months after surgery. The estimated cumulative incidence of BCRL in the overall cohort was 5.6% (95% CI, 5.2–5.9%) at 24 months, 8.1% (95% CI, 7.7–8.5%) at 48 months, and 12% (95% CI, 12–13%) at 144 months. The incidence of BCRL was markedly higher among patients who underwent ALND (1057 [28%]) compared to those who underwent SLNB (671 [4%]) (Supplementary Fig. 1).

**Fig. 1:**
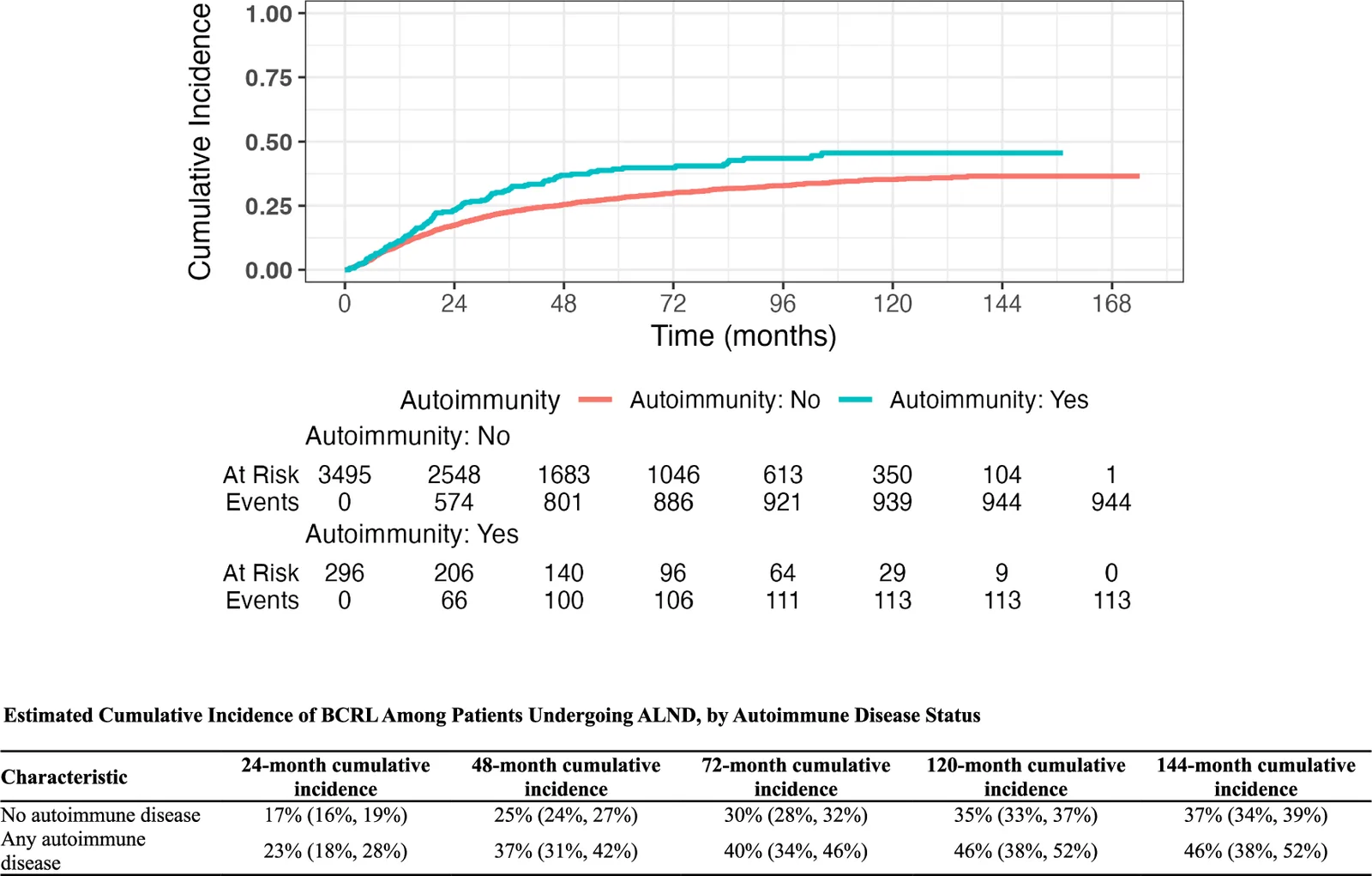
Kaplan–Meier plot for cumulative incidence of breast cancer-related lymphedema (BCRL) among patients undergoing axillary lymph node dissection (ALND), by autoimmune disease status. Kaplan–Meier curves showing the estimated cumulative incidence of BCRL among patients who underwent ALND with any autoimmune disease (blue) compared with those without autoimmune disease (red).

**Fig. 2:**
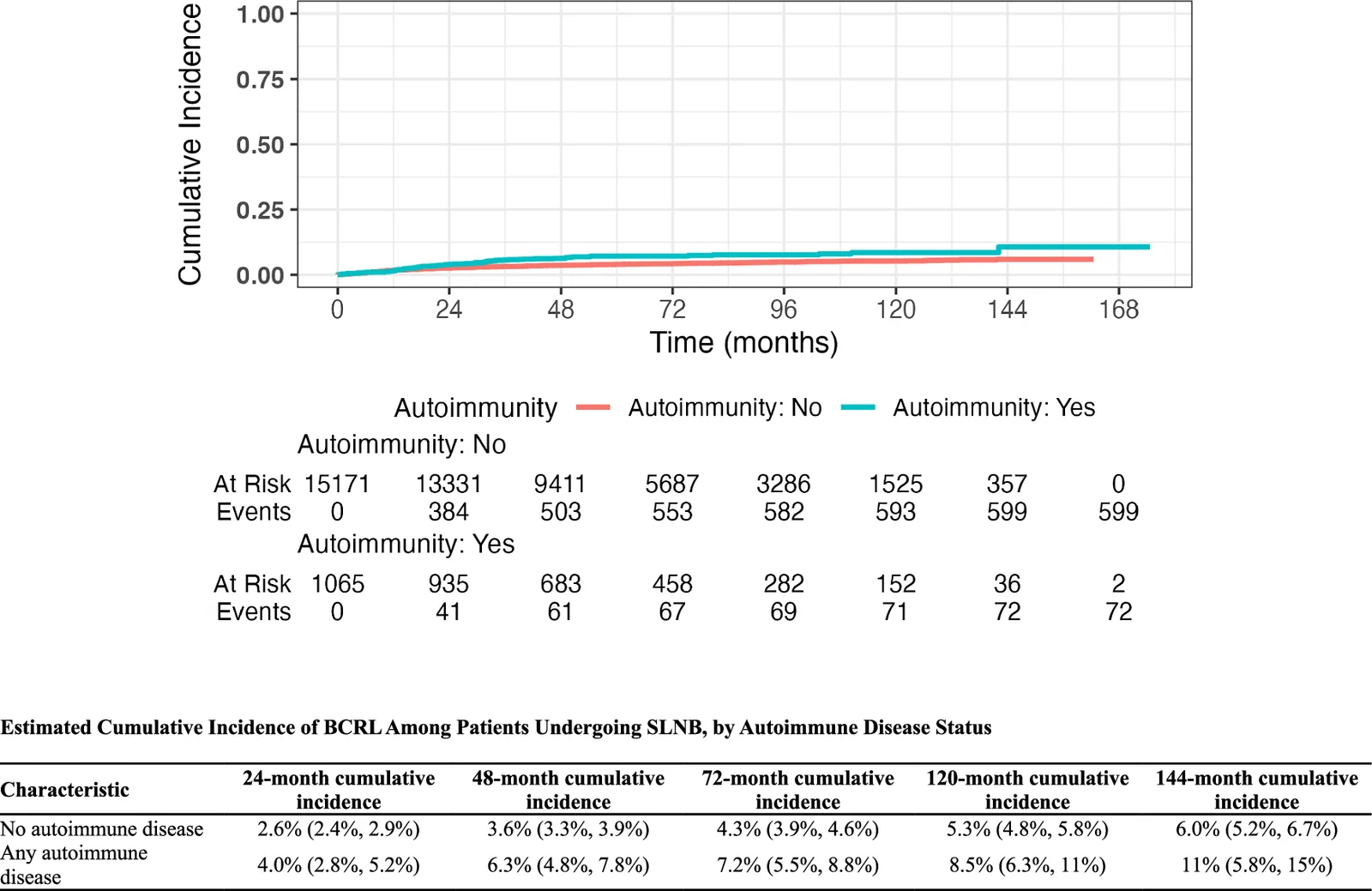
Kaplan–Meier plot for cumulative incidence of breast cancer-related lymphedema (BCRL) among patients undergoing sentinel lymph node biopsy (SLNB), by autoimmune disease status. Kaplan–Meier curves showing the estimated cumulative incidence of BCRL among patients who underwent SLNB with any autoimmune disease (blue) compared with those without autoimmune disease (red).

Preexisting autoimmune disease was present in 1361 (6.8%) patients, including 185 (11%) of those with BCRL and 1176 (6.4%) without (Table 2). The most common conditions among the cohort were RA (1.9%), autoimmune thyroiditis (1.3%), Graves’ disease (0.7%), and antiphospholipid syndrome (0.6%). Cumulative incidence of BCRL differed significantly by autoimmune status (Supplementary Fig. 2). Among patients undergoing ALND, autoimmune disease was associated with a substantially higher cumulative incidence of BCRL (Fig. 1). A similar, though less pronounced, difference was observed among patients undergoing SLNB (Fig. 2).

**Table 2:** Prevalence of autoimmune conditions overall and by breast cancer-related lymphedema (BCRL) diagnosis

Characteristic	Overall (*n* = 20,027)	BCRL (*n* = 1728)	No BCRL (*n* = 18,299)
Any autoimmune disease, No. (%)	1361 (6.8)	185 (11)	1176 (6.4)
Specific autoimmune conditions, No. (%)			
Rheumatoid arthritis	385 (1.9)	47 (2.7)	338 (1.8)
Autoimmune thyroiditis	262 (1.3)	43 (2.5)	219 (1.2)
Graves’ disease	140 (0.7)	23 (1.3)	117 (0.6)
Antiphospholipid syndrome	115 (0.6)	14 (0.8)	101 (0.6)
Celiac disease	95 (0.5)	10 (0.6)	85 (0.5)
Sjögren’s syndrome	85 (0.4)	9 (0.5)	76 (0.4)
Multiple sclerosis	84 (0.4)	13 (0.8)	71 (0.4)
Vitiligo	76 (0.4)	6 (0.3)	70 (0.4)
SLE	73 (0.4)	16 (0.9)	57 (0.3)
Systemic sclerosis (scleroderma)	51 (0.3)	11 (0.6)	40 (0.2)
Atopic dermatitis	37 (0.2)	8 (0.5)	29 (0.2)
Pernicious anemia	28 (0.1)	4 (0.2)	24 (0.1)
Dermatomyositis	15 (<0.1)	3 (0.2)	12 (<0.1)
Myasthenia gravis	17 (<0.1)	1 (<0.1)	16 (<0.1)
Primary biliary cholangitis	17 (<0.1)	1 (<0.1)	16 (<0.1)
Autoimmune hepatitis	7 (<0.1)	1 (<0.1)	6 (<0.1)
Polymyositis	6 (<0.1)	2 (0.1)	4 (<0.1)
Addison’s disease	6 (<0.1)	2 (0.1)	4 (<0.1)
Type I diabetes mellitus	2 (<0.1)	1 (<0.1)	1 (<0.1)
Crohn’s disease	0 (0)	0 (0)	0 (0)
Enteropathic arthropathies	0 (0)	0 (0)	0 (0)
Inflammatory bowel diseases	0 (0)	0 (0)	0 (0)
Juvenile arthritis	0 (0)	0 (0)	0 (0)
Primary sclerosing cholangitis	0 (0)	0 (0)	0 (0)
Psoriasis	0 (0)	0 (0)	0 (0)
Ulcerative colitis	0 (0)	0 (0)	0 (0)
Vasculitis	0 (0)	0 (0)	0 (0)

*SLE* systemic lupus erythematosus.

On univariate analysis, any autoimmune disease was significantly associated with increased BCRL risk (HR, 1.62; 95% CI, 1.39–1.89; *P* < 0.001) (Table 3). This association remained significant in multivariable analysis after adjustment for clinical and demographic factors (HR, 1.52; 95% CI, 1.30–1.77; *P* < 0.001). Several individual autoimmune conditions were also independently associated with elevated risk, including RA (HR, 1.54; 95% CI, 1.15–2.07; *P* = 0.004), autoimmune thyroiditis (HR, 1.40; 95% CI, 1.03–1.91; *P* = 0.031), Graves’ disease (HR, 1.59; 95% CI, 1.05–2.40; *P* = 0.028), multiple sclerosis (HR, 1.87; 95% CI, 1.08–3.22; *P* = 0.026), SLE (HR, 2.46; 95% CI, 1.47–4.14; *P* < 0.001), and systemic sclerosis (HR, 2.10; 95% CI, 1.16–3.81; *P* = 0.015).

**Table 3:** Univariate and multivariable Cox proportional hazards regression models for time to breast cancer-related lymphedema (BCRL)

	Univariate		Multivariate, any autoimmune disease		Multivariate, specific autoimmune diseases	
Characteristic	HR (95% CI)	*P* value	HR (95% CI)	*P* value	HR (95% CI)	*P* value
Age at surgery, per 1-year increase	0.98 (0.98, 0.98)	<0.001^b^	1.00 (0.99, 1.00)	0.2	1.00 (0.99, 1.00)	0.2
Sex						
Female	—		—			
Male	1.14 (0.70, 1.87)	0.6	0.99 (0.60, 1.63)	>0.9	0.99 (0.60, 1.63)	>0.9
Race/ethnicity						
White	—		—		—	
Asian	0.92 (0.76, 1.11)	0.4	0.84 (0.69, 1.01)	0.066	0.83 (0.69, 1.01)	0.058
Black	1.93 (1.69, 2.22)	<0.001^b^	1.33 (1.15, 1.53)	<0.001^b^	1.33 (1.15, 1.53)	<0.001^b^
Hispanic	1.46 (1.23, 1.72)	<0.001^b^	1.15 (0.97, 1.36)	0.12	1.15 (0.97, 1.37)	0.10
Other	1.63 (1.13, 2.34)	0.008^b^	1.27 (0.88, 1.83)	0.2	1.27 (0.88, 1.83)	0.2
Unknown	1.14 (0.90, 1.46)	0.3	1.04 (1.81, 1.33)	0.8	1.01 (0.79, 1.30)	>0.9
BMI, per 1-kg/m^2^ increase	1.02 (1.01, 1.03)	<0.001^b^	1.03 (1.02, 1.04)	<0.001^b^	1.03 (1.02, 1.04)	<0.001^b^
Smoking status						
Never smoker	—		—		—	
Current smoker	1.10 (0.82, 1.48)	0.5	1.03 (0.77, 1.38)	0.8	1.03 (0.77, 1.38)	0.8
Former smoker	0.74 (0.64, 0.85)	<0.001^b^	0.92 (0.79, 1.06)	0.2	0.92 (0.79, 1.06)	0.2
Unknown	1.00 (0.86, 1.16)	>0.9	1.05 (0.90, 1.22)	0.5	1.04 (0.90, 1.21)	0.6
Axillary surgery						
SLNB	—		—		—	
ALND	7.71 (7.00, 8.50)	<0.001^b^	4.01 (3.54, 4.53)	<0.001^b^	3.99 (3.53, 4.52)	<0.001^b^
Breast surgery						
BCS	—		—		—	
Mastectomy	2.38 (2.17, 2.62)	<0.001^b^	1.34 (1.18, 1.51)	<0.001^b^	1.33 (1.18, 1.51)	<0.001^b^
No breast surgery	10.2 (1.43, 72.5)	0.020^b^	1.99 (0.28, 14.3)	0.5	1.46 (0.20, 10.9)	0.7
Any chemotherapy						
No	—					
Yes	5.78 (5.05, 6.62)	<0.001^b^				
Chemotherapy timing						
None	—		—		—	
Adjuvant	4.12 (3.56, 4.76)	<0.001^b^	2.19 (1.86, 2.57)	<0.001^b^	2.19 (1.87, 2.57)	<0.001^b^
Neoadjuvant	8.80 (7.61, 10.2)	<0.001^b^	3.16 (2.66, 3.75)	<0.001^b^	3.19 (2.68, 3.79)	<0.001^b^
Unknown	11.1 (8.11, 15.1)	<0.001^b^	5.09 (3.70, 6.99)	<0.001^b^	5.21 (3.79, 7.18)	<0.001^b^
Any radiotherapy						
No	—		—		—	
Unknown	0.58 (0.36, 0.94)	0.026^b^	1.06 (0.65, 1.73)	0.8	1.06 (0.65, 1.72)	0.8
Yes	2.69 (2.36, 3.07)	<0.001^b^	1.91 (1.64, 2.22)	<0.001^b^	1.91 (1.64, 2.22)	<0.001^b^
Nodal radiotherapy						
No	—					
Unknown	0.36 (0.23, 0.57)	<0.001^b^				
Yes	3.63 (3.28, 4.01)	<0.001^b^				
Any endocrine therapy						
No	—		—		—	
Unknown	0.41 (0.18, 0.92)	0.031^b^	0.83 (0.37, 1.87)	0.7	0.84 (0.37, 1.88)	0.7
Yes	0.83 (0.74, 0.92)	<0.001^b^	0.94 (0.84, 1.05)	0.3	0.94 (0.84, 1.05)	0.3
Any autoimmune disease	1.62 (1.39, 1.89)	<0.001^b^	1.52 (1.30, 1.77)	<0.001^b^		
Rheumatoid arthritis	1.43 (1.07, 1.91)	0.015^b^			1.54 (1.15, 2.07)	0.004^b^
Autoimmune thyroiditis	1.80 (1.33, 2.44)	<0.001^b^			1.40 (1.03, 1.91)	0.031^b^
Graves’ disease	1.92 (1.27, 2.90)	0.002^b^			1.59 (1.05, 2.40)	0.028^b^
Sjögren’s syndrome	1.28 (0.67, 2.47)	0.5			1.15 (0.58, 2.27)	0.7
Multiple sclerosis	1.98 (1.15, 3.42)	0.014^b^			1.87 (1.08, 3.22)	0.026^b^
Vitiligo	0.87 (0.39, 1.95)	0.7			0.74 (0.33, 1.66)	0.5
SLE	2.49 (1.52, 4.07)	<0.001^b^			2.46 (1.47, 4.14)	<0.001^b^
Systemic sclerosis	2.24 (1.24, 4.06)	0.008^b^			2.10 (1.16, 3.81)	0.015^b^
Antiphospholipid syndrome	1.40 (0.83, 2.37)	0.2			1.11 (0.65, 1.90)	0.7
Celiac disease	1.23 (0.66, 2.28)	0.5			1.04 (0.56, 1.94)	>0.9
Pernicious anemia	1.73 (0.65, 4.62)	0.3			1.76 (0.66, 4.73)	0.3
Atopic dermatitis	2.44 (1.22, 4.88)	0.012^b^			1.69 (0.84, 3.39)	0.14
Primary biliary cholangitis^a^	0.64 (0.09, 4.54)	0.7				
Myasthenia gravis^a^	0.75 (0.11, 5.34)	0.8				
Dermatomyositis^a^	2.21 (0.71, 6.87)	0.2				
Polymyositis^a^	4.01 (1.00, 16.0)	0.050				
Addison’s disease^a^	4.89 (1.22, 19.6)	0.025^b^				
Autoimmune hepatitis^a^	1.85 (0.26, 13.1)	0.5				
Type I diabetes mellitus^a^	6.32 (0.89, 44.9)	0.065				

*ALND* axillary lymph node dissection; *BCS*, breast-conserving surgery, *BMI* body mass index, *CI* confidence interval, *HR* hazard ratio, *SLE* systemic lupus erythematosus, *SLNB* sentinel lymph node biopsy.

^a^ Indicates disease prevalence <0.1% in study cohort.

^b^ Statistically significant values.

Additional factors associated with BCRL development included Black race (HR, 1.33; 95% CI, 1.15–1.53; *P* < 0.001) and higher BMI (HR, 1.03 per 1-kg/m^2^ increase; 95% CI, 1.02–1.04; *P* < .001). Surgical and treatment-related factors independently associated with BCRL were ALND (HR, 4.01; 95% CI, 3.54–4.53; *P* < 0.001), mastectomy (HR, 1.34; 95% CI, 1.18–1.51; *P* < 0.001), adjuvant chemotherapy (HR, 2.19; 95% CI, 1.86-2.57; *P* < .001), neoadjuvant chemotherapy (HR, 3.16; 95% CI, 2.66–3.75; *P* < 0.001), and any radiotherapy (HR, 1.91; 95% CI, 1.64–2.22; *P* < 0.001) (Table 3).

## Discussion

In this large, single-institution case-control study of more than 20,000 patients who underwent treatment for breast cancer, preexisting autoimmune disease was independently associated with a significantly increased risk of developing BCRL. Several autoimmune conditions, including SLE, systemic sclerosis, multiple sclerosis, RA, Graves’ disease, and autoimmune thyroiditis, were associated with a more than 40% increased risk of lymphedema, with some demonstrating a more than twofold elevation in risk. These findings provide the most comprehensive clinical evidence to date that immune dysregulation contributes to individual susceptibility to secondary lymphedema after breast cancer treatment.

Previous research examining risk factors for BCRL has largely emphasized treatment-related predictors, including extent of axillary surgery, radiotherapy, and chemotherapy, as well as patient factors such as elevated BMI and Black race.^8,10,11,24,25^ The present analysis confirms the strong effect of these established risk factors while identifying autoimmunity as a novel, patient-level determinant of risk. Importantly, autoimmune disease remained associated with higher lymphedema incidence across both ALND and SLNB cohorts, indicating that immune dysregulation may increase vulnerability to lymphatic injury even after limited axillary surgery. To our knowledge, this is the first large-scale study to systematically evaluate a broad spectrum of autoimmune diseases and their association with BCRL within a diverse breast cancer population. The associations observed are consistent with preclinical and translational research implicating immune dysregulation, particularly chronic inflammation, macrophage infiltration, and Th2-driven fibrosis, in lymphatic injury and disease progression.^26^

The link between autoimmunity and lymphedema is likely multifactorial, reflecting shared inflammatory pathways and the close interplay between the immune and lymphatic systems. Both lymphedema and many autoimmune diseases are characterized by chronic inflammation that promotes progressive tissue fibrosis. In lymphedema, lymphatic fluid stasis initiates a cycle of inflammation and fibroadipose replacement of functional lymphatic vessels, further impairing lymphatic drainage.^13,27^ Studies from our group and others have shown that Th2-biased immune responses play a central role in this process. Differentiation of CD4^+^ T cells into Th2 subsets, driven by interleukin (IL)-4 and IL-13, promotes tissue fibrosis, lymphatic leakiness, and impaired lymphatic function.^14,15,27–31^ These mechanisms parallel the immune signatures observed in many autoimmune diseases, such as atopic dermatitis, which are fundamentally regulated by Th2 inflammatory responses.^14,32^ Transforming growth factor-beta 1, a potent profibrotic and anti-lymphangiogenic cytokine, is elevated in BCRL and is a hallmark of systemic sclerosis.^29,33^ Similarly, infiltrating macrophages and activated keratinocytes in lymphedematous skin produce Th2-inducing cytokines, mirroring immune dysregulation in autoimmune dermatologic diseases.^14,34,35^ Together, these shared inflammatory and profibrotic pathways suggest that chronic immune activation in autoimmune conditions may compromise lymphatic repair and resilience, providing a biologic basis for the increased risk of secondary lymphedema.

Two non-mutually exclusive mechanisms may explain the association between autoimmunity and increased lymphedema risk. The first is that chronic systemic inflammation in autoimmune diseases may damage lymphatic endothelial cells, leading to increased permeability, oxidative stress, and fibrosis, thus impairing baseline lymphatic function and regenerative capacity. When superimposed injury from surgery or radiation occurs, the lymphatic system may no longer be able to restore lymphatic integrity.^36^ It is also possible that inflammation resulting from autoimmune diseases may directly remodel the lymphatic vasculature, leading to functional changes through cytokine-mediated injury and immune cell infiltration, resulting in contractile dysfunction, valvular insufficiency, and fibrosis of lymphatic collectors. These processes converge to create a lymphatic environment that is less resilient to injury, with decreased lymphatic reserve and thus more prone to chronic failure.

Emerging clinical and experimental evidence across multiple autoimmune diseases supports these proposed mechanisms. In RA, early inflammation promotes compensatory lymphangiogenesis and enhanced lymphatic pumping, but chronic disease leads to lymphatic collapse, diminished contraction amplitude, and reduced lymph node drainage.^37^ Near-infrared imaging in patients with RA and experimental animal models shows decreased lymphatic clearance and shortened vessel length despite preserved contraction frequency; these findings parallel the functional impairments observed in advanced lymphedema and illustrate how recurrent inflammation can progressively exhaust lymphatic transport capacity.^38^

In addition to inflammation-driven functional impairment, other autoimmune diseases illustrate how persistent immune activation can directly alter lymphatic endothelial identity and vessel architecture. In systemic sclerosis, lymphatic endothelial cells undergo endothelial-to-mesenchymal transition, leading to progressive lymphatic fibrosis and impaired vessel contractility, a process that mirrors the fibrotic remodeling seen in secondary lymphedema.^39,40^ Similarly, in autoimmune valvular carditis, persistent inflammation drives aberrant vascular endothelial growth factor (VEGF) receptor 3-dependent lymphangiogenesis, producing expanded but nonfunctional and fibrotic lymphatic vessels; inhibition of VEGF receptor 3 or VEGF-C signaling reverses this pathology.^41^ Collectively, these findings illustrate how autoimmune inflammation can both impair lymphatic function through inflammatory flares and directly alter lymphatic phenotype through endothelial reprogramming, producing a chronically fibrotic and transport-deficient state characteristic of secondary lymphedema.

The differential hazard ratios observed in this study reflect this mechanistic hierarchy: conditions with the most direct structural lymphatic consequences, particularly SLE and systemic sclerosis, conferred the greatest elevations in risk, while conditions whose primary pathology is less directly lymphotropic conferred more modest but still significant increases, suggesting a spectrum of lymphatic vulnerability rather than a binary effect. The elevated risk in Graves’ disease may reflect an additional contribution from TSH-receptor autoantibody-driven fibroblast activation and glycosaminoglycan deposition in extrathyroidal tissues, increasing interstitial fluid burden and potentially reducing lymphatic reserve.^22^ Importantly, the composite autoimmune disease variable spans this heterogeneous spectrum of conditions; this heterogeneity likely attenuates the overall composite hazard ratio, and future studies with sufficient power should examine disease category-specific associations.

Although autoimmunity can compromise lymphatic structure and function, the converse is also true: lymphatic dysfunction may perpetuate or amplify autoimmune-mediated inflammation. Lymphatics are essential for immune surveillance, antigen clearance, and maintenance of peripheral tolerance.^22^ Impaired lymphatic transport leads to inefficient antigen drainage, prolonged antigen exposure within tissues, and sustained autoantibody formation.^22,38^ Experimental models demonstrate that mice lacking dermal lymphatics develop impaired humoral immunity, loss of tolerance, and spontaneous autoimmunity with autoantibody deposition.^42^ Similarly, in SLE, ultraviolet radiation (UVR)-induced lymphatic flow dysfunction worsens cutaneous inflammation and augments B- and T-cell activation in draining lymph nodes.^23^ These reciprocal interactions suggest that lymphatic failure not only results from autoimmune-mediated inflammation but also drives its persistence, establishing a self-perpetuating cycle of immune and lymphatic dysfunction.

The clinical implications of these findings are substantial. Autoimmune disease status should be incorporated into preoperative counseling to guide individualized lymphedema risk assessment before surgery and radiation. Those with preexisting autoimmune conditions may benefit from closer surveillance, early referral to physical therapy, and prophylactic strategies such as compression therapy and immediate lymphatic reconstruction.^43^ Identifying these high-risk individuals could also help select patients most likely to benefit from early surgical interventions aimed at improving lymphatic function, including lymphovenous bypass or vascularized lymph node transplant.^44^ Mechanistic overlap further raises the possibility for therapeutic crossover. Manual lymphatic drainage, widely used for lymphedema, has been shown to improve lymphatic flow and reduce UVR-induced skin inflammation, splenomegaly, and autoantibody titers in SLE.^23^ Similarly, several anti-inflammatory and immunomodulatory drugs used for autoimmune diseases, including IL-4/IL-13–neutralizing antibodies, calcineurin inhibitors such as tacrolimus, and FDA-approved multiple sclerosis therapies (fingolimod, teriflunomide), have demonstrated beneficial effects in preclinical lymphedema models and, in some cases, early-phase clinical trials.^14,30,34,45–48^ These observations suggest that interventions targeting shared inflammatory pathways may mitigate BCRL risk and progression in high-risk patients.

The potential contribution of autoreactive immune mechanisms may also be relevant to primary lymphedema, which exhibits similar pathophysiologic features to secondary forms and occurs more frequently in females around puberty, a period that coincides with the onset of many autoimmune diseases.^49–51^ This parallel suggests that immune dysregulation may contribute to disease susceptibility across both inherited and acquired forms of lymphatic dysfunction. Future research might also explore whether biomarkers of systemic immune activation can predict lymphedema onset, enabling earlier detection and prevention. Finally, given the chronic and progressive nature of both autoimmune disease and lymphedema, longitudinal studies are needed to determine how disease activity, flares, and long-term treatment exposures influence lymphedema risk over time.

This study should be interpreted in the context of several limitations. Its retrospective design and reliance on ICD coding for both lymphedema and autoimmune disease may have introduced misclassification bias. For BCRL, ICD-based ascertainment likely captures clinically recognized cases while under-detecting milder or subclinical disease, primarily affecting estimates of absolute incidence rather than relative risk. For autoimmune disease, ICD-based ascertainment may result in non-differential misclassification that would be expected to bias associations toward the null, suggesting the observed hazard ratios may be conservative estimates. Although these ICD-based algorithms have not been formally validated in analogous breast cancer survivorship cohorts, similar approaches have been widely used in large retrospective cohort studies and represent the most feasible, scalable method for this cohort.^11,52,53^ Although coding accuracy at our institution is high, subclinical or undiagnosed cases may have been missed, and chart review could not be performed for the entire cohort. Notably, several immune-mediated conditions (e.g., Crohn’s disease, ulcerative colitis, psoriasis) were absent from the cohort, suggesting incomplete exposure ascertainment for some conditions. The single-institution setting may limit generalizability to more diverse patient populations and treatment contexts. We lacked detailed data on autoimmune disease activity, severity, and immunosuppressive therapy use; immunosuppressive therapies may exert bidirectional effects on BCRL risk, and the observed associations may reflect a combination of disease biology and treatment effects. Similarly, we could not capture lymphedema stage, severity, or use of disease-modifying treatments, limiting assessment of disease trajectory or treatment response. Hazard ratio estimates for rare autoimmune conditions should be interpreted with caution, given small event counts and wide confidence intervals. Cumulative incidence estimates were derived using Kaplan–Meier methods, which treat death as a non-informative censoring event; if patients who die are systematically at higher risk of lymphedema than those who remain under observation, this assumption may be violated, and cumulative incidence could be modestly overestimated relative to a formal competing risks framework. Finally, although we adjusted for established demographic and treatment-related confounders, residual confounding from unmeasured variables remains possible. Prospective, multicenter studies incorporating objective limb measurements and standardized autoimmune disease characterization are needed to validate and extend these findings.

In conclusion, preexisting autoimmune disease is independently associated with increased risk factor for BCRL, with some conditions conferring more than a twofold increase in risk. These findings support the integration of autoimmune disease status into lymphedema risk assessment, patient counseling, and surveillance strategies. Recognition of this association may enable earlier detection and intervention in high-risk patients and provide a rationale for future research on the mechanistic links between immune dysregulation and lymphatic dysfunction. Furthermore, understanding that autoreactive immune reactions may contribute to disease pathophysiology may help guide future investigations into the underlying mechanisms of BCRL and the development of targeted preventive or therapeutic approaches.

## Methods

### Study population

This study was approved by the Institutional Review Board at Memorial Sloan Kettering Cancer Center (IRB #16-1038), with a waiver of informed consent due to its retrospective design. Eligible patients were 18 years or older, had a diagnosis of breast cancer, and underwent ALND and/or sentinel lymph node biopsy (SLNB) at the study institution between January 2010 and January 2021. Patients with missing or incomplete data were excluded.

### Study variables

Demographic variables collected for analysis included age at axillary surgery, sex, self-reported race and ethnicity, body mass index (BMI), and smoking status. Clinical variables included type of axillary surgery (ALND or SLNB), type of breast surgery (breast-conserving surgery or mastectomy), any chemotherapy, timing of chemotherapy (neoadjuvant or adjuvant), any radiotherapy, nodal radiotherapy, and any endocrine therapy. Patients who underwent both SLNB and ALND were classified in the ALND group.

Autoimmune conditions were identified through a structured query of International Classification of Diseases (ICD)-9 and ICD-10 codes (Supplementary Table 1). Specific conditions included Addison’s disease, antiphospholipid syndrome, atopic dermatitis, autoimmune hepatitis, autoimmune thyroiditis, celiac disease, Crohn’s disease, dermatomyositis, enteropathic arthropathies, Graves’ disease, inflammatory bowel disease, juvenile arthritis, multiple sclerosis, myasthenia gravis, pernicious anemia, polymyositis, primary biliary cholangitis, primary sclerosing cholangitis, psoriasis, RA, Sjögren’s syndrome, SLE, systemic sclerosis, type 1 diabetes mellitus, ulcerative colitis, vasculitis, and vitiligo.

The primary outcome was the development of BCRL, identified using ICD-9 and ICD-10 codes (I97.2, I89.0, I97.210, 457, and 457.1). Follow-up duration was calculated in months from the date of surgery to the last recorded clinic visit or death, whichever occurred first. Time from surgery to BCRL diagnosis was also recorded.

### Statistical analysis

Patient demographics, clinical characteristics, and autoimmune conditions were summarized overall and by BCRL diagnosis. Continuous variables were reported as medians with interquartile ranges (IQRs), and categorical variables were reported as counts and proportions. Univariate and multivariable Cox proportional hazards models were used to evaluate associations between autoimmune disease and BCRL development, with results presented as hazards ratios (HRs) and 95% confidence intervals (CIs). To account for potential multicollinearity between the composite variable “any autoimmune disease” and individual autoimmune conditions, two separate multivariable Cox proportional hazards models were constructed, one for each variable set. Time to BCRL was defined as the interval from the date of axillary surgery to the date of lymphedema diagnosis. Patients without BCRL were censored at the date of last follow-up. Variables for multivariable models were selected a priori based on clinical relevance. Autoimmune conditions affecting fewer than 0.1% of the cohort were excluded from multivariable models to reduce numerical instability and avoid inflation of effect estimates from rare diseases; estimates for conditions near this threshold should be interpreted with caution, given small event counts and wide confidence intervals. Data were analyzed from May to August 2025. All analyses were conducted in R, version 4.4.1 (R Foundation for Statistical Computing). A two-sided *P* value <0.05 was considered statistically significant.

## Supplementary information

Supplementary Material

## Data Availability

The datasets generated and/or analyzed during the current study are not publicly available due to institutional review board (IRB) restrictions and the inclusion of protected health information from a single-institution clinical cohort, which limit public data sharing under HIPAA regulations, but are available from the corresponding author on reasonable request.
