# Supplementary material for: Association between autoimmune diseases and risk of developing breast cancer-related lymphedema
Source: NPJ Autoimmun. 2026 Oct 24;1(1):5. doi: 10.1038/s44483-026-00004-3 (PMC13695572; doi:10.1038/s44483-026-00004-3)
Supplement: Supplementary file 1 [file 44483_2026_4_MOESM1_ESM.pdf]

Supplementary Material

Supplementary Table 1. International Classification of Diseases (ICD) Codes Used to Identify Autoimmune Diseases in the Study Cohort

Supplementary Figure 1. Kaplan-Meier Plot for Cumulative Incidence of Breast Cancer-Related Lymphedema (BCRL) by Axillary Surgery Type

Supplementary Figure 2. Kaplan-Meier Plot for Cumulative Incidence of Breast Cancer-Related Lymphedema (BCRL) by Autoimmune Disease Status

Supplementary Table 1. International Classification of Diseases (ICD) Codes Used to Identify Autoimmune Diseases in the Study Cohort

Condition	ICD-9 Codes	ICD-10 Codes
Addison's disease	255.4	E27.1, E27.2
Celiac disease	579.0	K90.0
Dermatomyositis	710.3	M33.00, M33.0, M33.02, M33.03, M33.1, M33.10, M33.12, M33.13, M33.19, M36.0, M36.9, M33.90, M33.91, M33.99
Polymyositis	710.4	M33.2, M33.20, M33.29
Graves' disease	242.0	E05.0, E05.00
Autoimmune thyroiditis	245.2	E06.3
Multiple sclerosis	340	G35
Myasthenia gravis	358.00, 358.01	G70.00, G70.01, G70.2
Pernicious anemia	281.0	D51.0
Psoriasis	696	L40
Rheumatoid arthritis	714.0, 714.1, 714.2, 714.4, 714.8, 714.9	M05, M06, M06.9, M05.9
Juvenile arthritis	714.3	M08
Sjögren's syndrome	710.2	M35.00, M35.09
Systemic lupus erythematosus	710.0	M32
Type I diabetes mellitus	250-250.99	E10
Systemic sclerosis	701.0, 710.1	M34
Antiphospholipid syndrome	289.81	D68.61
Atopic dermatitis	691, 691.8	L20, L20.8, L20.89, L20.9
Vitiligo	709.01	L80
Crohn's disease	555	K50
Ulcerative colitis	556	K51
Vasculitis	446	M31
Autoimmune hepatitis	571.42	K75.4
Primary biliary cholangitis	571.6	K74.3
Primary sclerosing cholangitis	—	K83.01
Inflammatory bowel diseases	555	—
Enteropathic arthropathies	713.1	M07

Supplementary Figure 1. Kaplan-Meier Plot for Cumulative Incidence of Breast Cancer-Related Lymphedema (BCRL) by Axillary Surgery Type

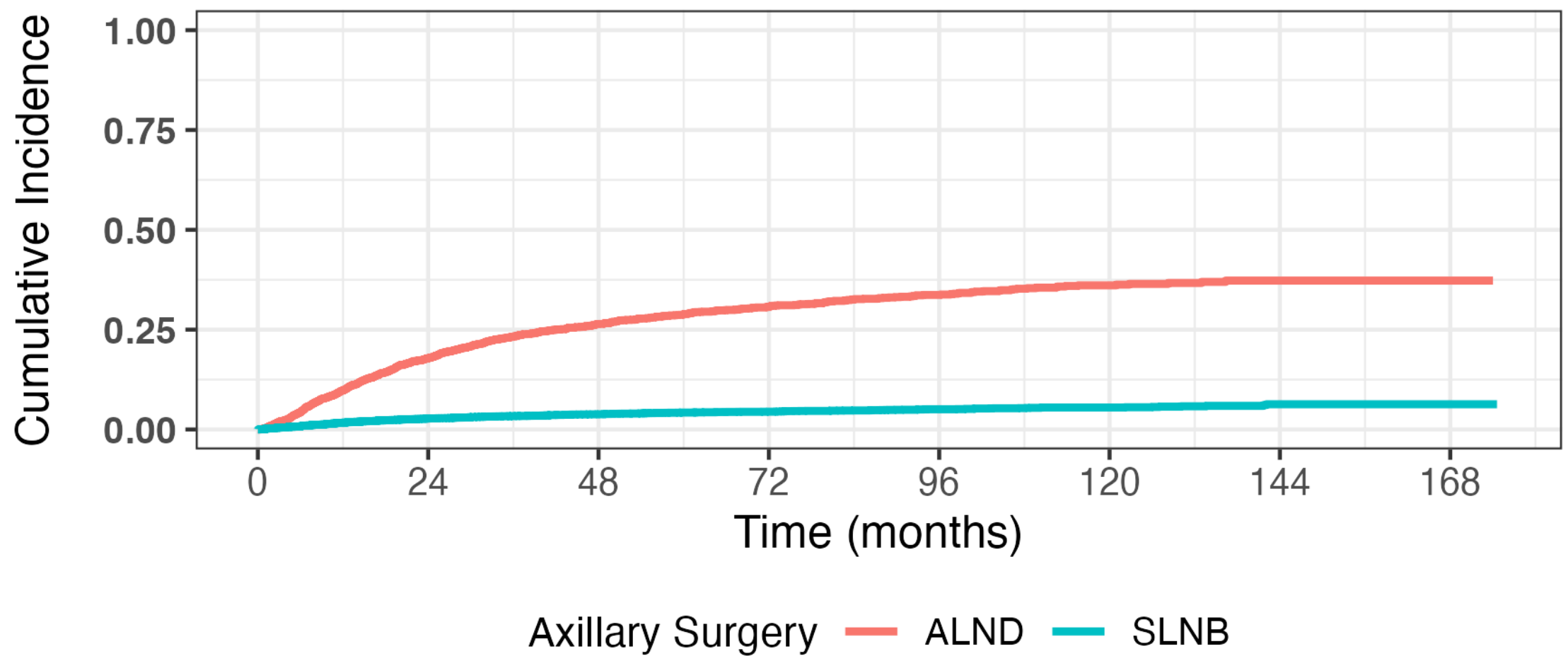

**ALND**								
At Risk	3791	2754	1823	1142	677	379	113	1
Events	0	640	901	992	1032	1052	1057	1057
**SLNB**								
At Risk	16236	14266	10094	6145	3568	1677	393	2
Events	0	425	564	620	651	664	671	671

Estimated Cumulative Incidence of BCRL by Axillary Surgery Type

Characteristic	24-month cumulative incidence	48-month cumulative incidence	72-month cumulative incidence	120-month cumulative incidence	144-month cumulative incidence
ALND	18% (17%, 19%)	26% (25%, 28%)	31% (29%, 32%)	36% (34%, 38%)	37% (35%, 40%)
SLNB	2.7% (2.5%, 3.0%)	3.8% (3.5%, 4.1%)	4.5% (4.1%, 4.8%)	5.5% (5.0%, 6.0%)	6.3% (5.5%, 7.1%)

Kaplan-Meier curve displaying the estimated cumulative incidence of BCRL by ALND (red) versus SLNB (blue).

Supplementary Figure 2. Kaplan-Meier Plot for Cumulative Incidence of Breast Cancer-Related Lymphedema (BCRL) by Autoimmune Disease Status

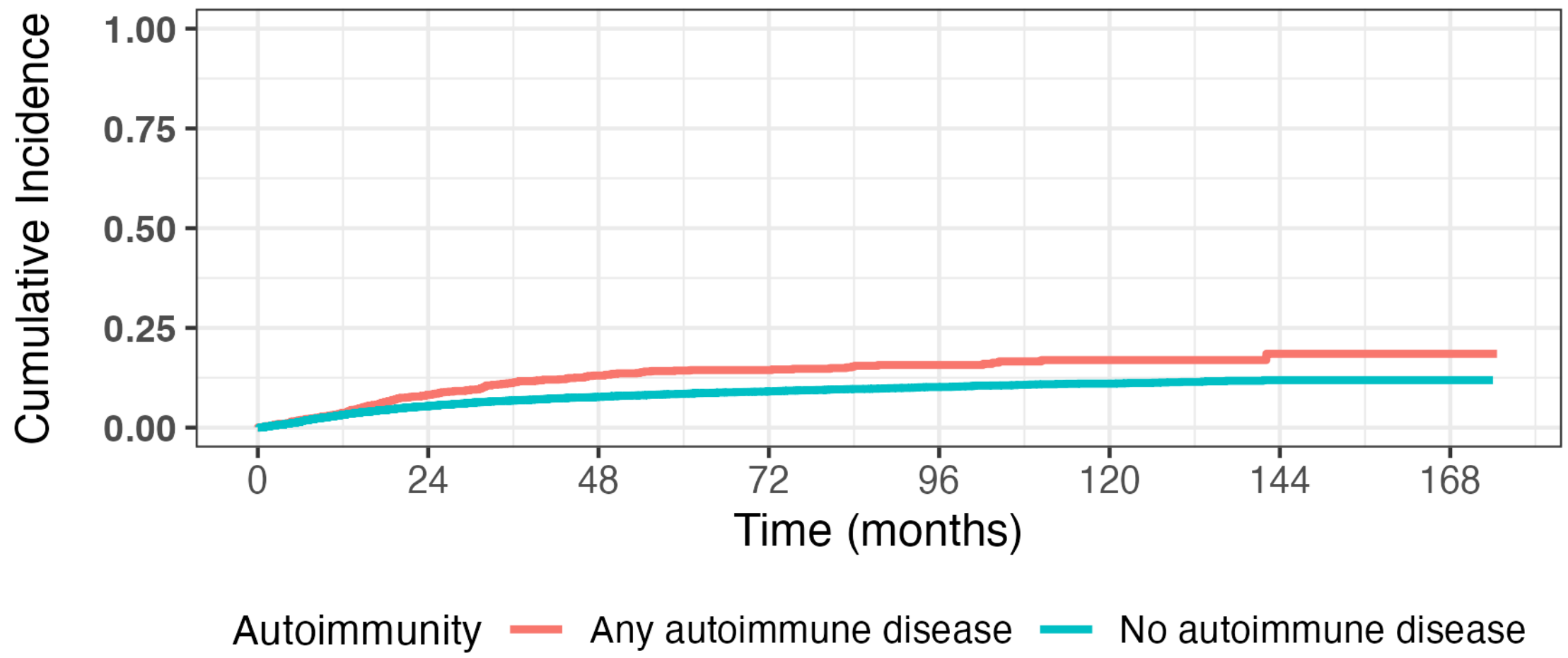

Any autoimmune disease

	0	24	48	72	96	120	144	168
At Risk	1361	1141	823	554	346	181	45	2
Events	0	107	161	173	180	184	185	185

No autoimmune disease

	0	24	48	72	96	120	144	168
At Risk	18666	15879	11094	6733	3899	1875	461	1
Events	0	958	1304	1439	1503	1532	1543	1543

Estimated Cumulative Incidence of BCRL by Autoimmune Disease Status

**Characteristic**	**24-month cumulative incidence**	**48-month cumulative incidence**	**72-month cumulative incidence**	**120-month cumulative incidence**	**144-month cumulative incidence**
Any autoimmune disease	8.2% (6.7%, 9.7%)	13% (11%, 15%)	14% (12%, 16%)	17% (14%, 19%)	18% (15%, 22%)
No autoimmune disease	5.4% (5.0%, 5.7%)	7.7% (7.3%, 8.1%)	9.1% (8.6%, 9.5%)	11% (10%, 12%)	12% (11%, 13%)

Kaplan-Meier curve displaying the estimated cumulative incidence of BCRL among patients with any autoimmune disease (red) compared with those without autoimmune disease (blue).
